# Supplementary material for: Mature adipocytes inhibit differentiation of myogenic cells but stimulate proliferation of fibro-adipogenic precursors derived from trout muscle in vitro
Source: Sci Rep. 2024 Jul 16;14:16422. doi: 10.1038/s41598-024-67152-0 (PMC11252293; doi:10.1038/s41598-024-67152-0)
Supplement: Supplementary file 1 — Supplementary Information 1. [file 41598_2024_67152_MOESM1_ESM.docx]

**Supplemental figure S1. Mononucleated muscle cells does not impact the morphology or activity of mature adipocytes *in vitro*.** (a) Bright field images of mature adipocytes (MA) extracted from perivisceral adipose tissue on day 1 before coculture (left)(n=22) or on day 4 cultured alone (right, top) (n=22) or with mononucleated muscles cells (MA + MMC) (right, bottom) (n=28). No morphological changes are visible. (b) Comparison of mature adipocytes intracellular activity, reflecting cellular viability, using the calcein AM dye. The percentage of strongly labeled cells shows a decrease in activity over time (D1 to D4), which is not impacted by the presence or absence of MMC (ScheirerRayHare: Condition p=0.83; Day p=0.0014, interaction p=0.86). Significance levels: ns (no significance), ** (p < 0.01).
