# Supplementary figures and images for: Mature adipocytes inhibit differentiation of myogenic cells but stimulate proliferation of fibro-adipogenic precursors derived from trout muscle in vitro
Source: Sci Rep. 2024 Jul 16;14:16422. doi: 10.1038/s41598-024-67152-0 (PMC11252293; doi:10.1038/s41598-024-67152-0)

**a**

Day 1

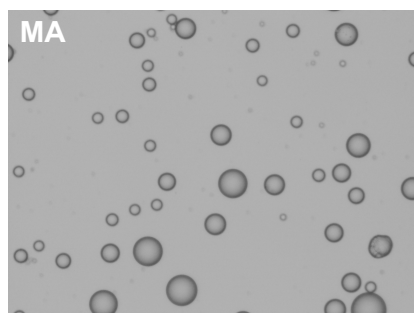

Day 4

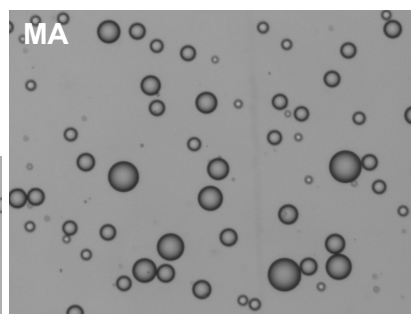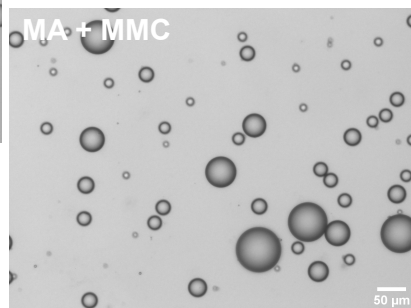**b**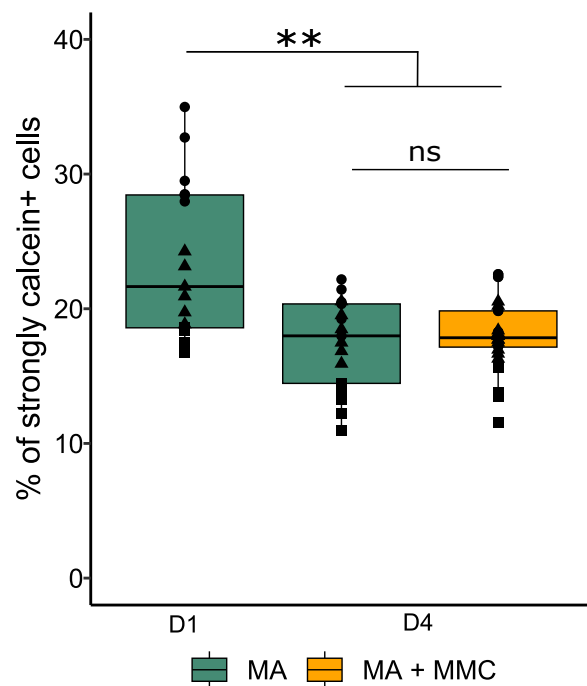

Supplement: Supplementary file 2 — Supplementary Information 2. [file 41598_2024_67152_MOESM2_ESM.pdf]
